# Antiretroviral Therapy Adherence and Clinic Attendance Over Time Among People in Argentina Living with HIV and Lost to Care

**DOI:** 10.1007/s12529-025-10356-z

**Published:** 2025-02-24

**Authors:** Omar Sued, Violeta J. Rodriguez, Stephen M. Weiss, Maria Luisa Alcaide, Diego Cecchini, Pedro Cahn, Isabel Cassetti, Chloe J. Kaminsky, Deborah L. Jones, The COPA Study Group

**Affiliations:** 1 Fundación Huésped, Buenos Aires, Argentina; 2 Department of Psychiatry and Behavioral Sciences, University of Miami Miller School of Medicine, 1440 Soffer Clinical Research Building, 1120 NW 14Th Street, Miami, FL 33136 USA; 3 Division of Infectious Diseases, Department of Medicine, University of Miami Miller School of Medicine, Miami, FL USA; 4 Hospital General de Agudos Dr. Cosme Argerich, Buenos Aires, Argentina; 5 Helios Salud, Buenos Aires, Argentina

**Keywords:** Argentina, HIV care, Adherence, Motivation, Behavioral intervention

## Abstract

**Background:**

Although Argentina provides access to no cost HIV care, treatment adherence and retention in care remain suboptimal. This study aimed to explore factors associated with self-reported adherence and appointment attendance over time.

**Method:**

Participants (*N* = 360) were people living with HIV (PLWH) that were lost to care (i.e., three missed pharmacy pickups in the last 6 months, or had not attended a physician visit in the last 12 months). Participants were recruited from seven HIV clinics in four urban centers in Argentina and re-engaged in care. Demographic variables, predictors, i.e., alcohol use, self-efficacy, motivation, patient-provider communication, insurance type (private/public), and outcomes, i.e., missed infectious disease (ID) specialist appointments, other missed clinic and lab appointments, and self-reported adherence were assessed over 2 years. A logistic regression and Poisson regression model within a generalized linear mixed model framework was used to analyze the association between predictors, treatment adherence outcomes, and interactions with time.

**Results:**

Following re-engagement in care, increased alcohol use was associated with lower odds of antiretroviral therapy adherence over time, increased odds of missing ID specialist appointments, and missed clinic/lab appointments. Self-efficacy was associated with better medication adherence and fewer missed ID specialist appointments over time. Similarly, both motivation and patient/provider communication were associated with fewer missed ID specialist and clinic/lab appointments over time. Having private health insurance was also associated with less missed clinic/lab appointments.

**Conclusion:**

Findings suggest alcohol use reduction interventions could improve treatment outcomes in this population. Additionally, interventions targeting patient-provider communication and patient self-efficacy and motivation may enhance retention following re-engagement in care.

## Introduction

No cost treatment is available for individuals living with HIV in Argentina through the National AIDS Program, and all people living with HIV (PLWH) needing healthcare can receive services through the public healthcare system [1]. Persons currently employed receive HIV care and treatment at no cost through the social security subsector (Obras Sociales) provided by employers. It is estimated that 60% of people living in Argentina receive healthcare coverage through Obras Sociales [2]. Those who are unemployed in Argentina are entitled to universal healthcare including free HIV treatment through the public system, regardless of nationality or immigration status. Any person may obtain healthcare through the private subsector by their own contribution in addition to or as an alternative to the public healthcare system [2].

Access to free HIV treatment has supported Argentine progress towards the UNAIDS goal to end AIDS by 2030 through implementation of the “Treatment for all: 95–95-95” plan, whereby 95% of PLWH will be aware of their status, antiretroviral therapy (ART) will be underway for 95% of those diagnosed, and 95% of those on ART will achieve an undetectable viral load [3]. Yet, despite ready availability of treatment, Argentina lags behind the 95% goal; 83.5% of diagnosed PLWH are on ART [4] and 16.5% of PLWH in Argentina remain untreated. Annually, there are 6500 new diagnoses and 1400 deaths related to HIV, and an estimated 126,000 people are living with HIV in Argentina [5]. Local data on the HIV continuum of care indicates gaps in early engagement in care and patient retention [5].

PLWH who are hard to engage, treat, and retain in treatment may be experiencing contextual factors, e.g., the deleterious effects of alcohol use, that can worsen the prognosis of HIV [6, 7]. The association between alcohol use and poor ART adherence has been well-documented and interventions targeting alcohol use may help to maintain adherence and the achievement of an undetectable HIV viral load. Studies assessing the efficacy of interventions for PLWH found that those with a strong focus on alcohol not only reduced alcohol consumption, but also increased adherence and condom use, which may reduce HIV and STD transmission [8]. Though alcohol use has been associated with poor ART adherence, alcohol use may fluctuate over time. These dynamic longitudinal associations have not been as extensively investigated as cross-sectional associations [9].

The strategies to enhance patient-provider communication may also enhance HIV treatment adherence. Effective patient-provider communication has been associated with increased adherence and treatment engagement among patients living with HIV as well as with other chronic health conditions, globally [10]. Specific aspects of patient-provider communication that may increase retention and care and health seeking behaviors include increased patient engagement in decision-making, and provider empathy and respect [11, 12]. Patient-provider communication is also related to self-efficacy, and prior intervention research in the USA found self-efficacy to be pivotal to treatment adherence [13] and attendance [14]. Patient motivation has also been associated with ART adherence, but has been less studied in the context of retention and appointment attendance [15].

In addition to alcohol use, communication, self-efficacy, and motivation, clinic factors (i.e., private versus public clinics) are related to adherence and retention in care. Patients attending private clinics in Argentina subsidized by private insurance have achieved better treatment adherence and lower viral load in comparison with patients attending public clinics subsidized by public insurance [16]. The impact of the private versus public healthcare system on patients living with chronic health conditions has been investigated in Argentina and globally; overall, patients who attend private clinics or primary care tend to report more positive experiences and have greater access to follow-up services and necessary medications [17–19]. Understanding the impact of differing healthcare systems on HIV care and adherence among residents in Argentina may be important to further understand healthcare disparities, especially among minority populations living with HIV.

This study addressed adherence and visit attendance among individuals lost to care from the perspective of self-determination theory, which emphasizes autonomy, competence, and relatedness, from which were drawn the elements of self-efficacy, motivation, and communication [20]. Lost to care was here defined as having missed three pharmacy pickups in the last 6 months or had not attended a physician visit in the last 12 months. The study aimed to assess the associations between predictive factors (i.e., communication, self-efficacy, motivation, as well as alcohol use and insurance type) and treatment adherence (self-reported adherence, attendance at appointments with infectious disease specialists, and clinic or lab appointments) over 2 years. Moreover, this study examined the interactions of these predictors and how these associations changed over time. It was hypothesized that interactions between time and self-efficacy, motivation, communication, and alcohol use and insurance type would be associated with overall treatment adherence (i.e., medication adherence and clinic attendance).

## Methods

### Setting, Participants, and Procedures

Data for this study was drawn from Conexiones y Opciones Positivas en la Argentina 2 (COPA2), a cluster-randomized clinical trial of physician-delivered motivational interviewing that has been previously described [21]. Participants were enrolled and completed a baseline assessment between November 10, 2016 and March 19, 2018. Participants were followed up from October 19, 2018 to May 27, 2020. The data were accessed for analyses between March 11, 2022 and April 21, 2022. The primary study outcomes have been previously reported [22], and suggested that over time, physician-delivered motivational interviewing (MI) enhanced the patient-provider relationship, self-efficacy, and ART adherence, and reduced HIV viral load in patients disengaged from HIV care. Before any study procedures, recruitment, or data analysis, approval was obtained from the affiliated US Institutional Review Board (IRB) and IRBs with oversight of all Argentine sites. Before enrolment and provision of informed consent, participants were provided detailed information on study procedures and confidentiality of study data in the local language (Spanish).

Participants (*N* = 360) were patients living with HIV who were lost to care. Participants were recruited from seven clinics serving PLWH in four Argentine urban centers across the country. Eligible patients were (1) 18 years of age or older, (2) had been diagnosed with HIV for more than 6 months, (3) had a recent (≤ 3 months) viral load of > 500 copies/mL after ≥ 6 months of ART prescription, and (4) were lost to care (i.e., three missed pharmacy pickups in the last 6 months, or had not attended a physician visit in the last 12 months).

To enhance disclosure, reduce social desirability bias, and accommodate all literacy levels, an audio computer-assisted self-interview (ACASI) system was used to complete study measures. All materials had been translated into Spanish and adapted to the local context in previous research and pilot studies [23] in preparation for the current study. All assessment data were digitally transmitted to the US site without identifying information.

### Demographic Variables

Demographic variables included age, gender, nationality (participants from Argentina or not from Argentina), sexual orientation, and education (dichotomized into less than high school completion or high school and higher).

### Outcomes

#### Self-reported Antiretroviral Adherence Over Time

Self-reported adherence was assessed using a 10-point Visual Analogue Scale (VAS) with 0 representing none and 10 representing 100% (perfect adherence) in the past week [24, 25]. Adherence was dichotomized at 100%, such that less than 100% was considered non-adherent and 100% adherent, this definition having been previously utilized with this sample [23]. If a participant reported being prescribed more than one pill per day, they then completed the VAS for each pill they were prescribed (up to three medications), and the average VAS adherence for that timepoint was calculated by dividing the sum of the VAS scores by the number of medications reported. Self-reported adherence was again measured at 24 months.

#### Missed Infectious Disease Appointments Over Time

At each time point (baseline, 6, 12, 18, and 24 months), participants were asked how many times in the past 12 months they were scheduled to see their infectious disease specialist but did not attend their appointment and did not call to make a new appointment.

#### Missed Lab Appointments Over Time

Participants were asked how many times in the last 12 months they were advised by their physician to go to a lab or clinic outside of their infectious disease center but did not go and did not ask for a new appointment. This outcome is measured at each time point (baseline, 6, 12, 18, and 24 months).

### Predictors

#### Time

Each predictor (i.e., self-efficacy, communication, motivation for adherence, and alcohol and health insurance type) was measured and examined over the course of 24 months, once at baseline, and at 6, 12, 18, and 24 months post intervention.

#### Self-efficacy

The HIV Treatment Adherence Self-Efficacy Scale (HIV-ASES) is a 12-item scale that assesses participants’ perceived self-efficacy in maintaining adherence and integrating their treatment in their daily life despite obstacles [26]. Cronbach’s reliability coefficient for this scale was considered excellent (*α* = 0.92). Response scores ranged from 0 (Cannot do at all) to 10 (Completely certain can do). Mean self-efficacy scores were used for analysis at each time point.

#### Alcohol

The Alcohol Use Disorders Identification Test (AUDIT, Spanish) uses a 10-item assessment of alcohol consumption, including frequency of consumption, binge drinking, and alcohol-related issues [27]. Cronbach’s reliability coefficient for this scale was considered acceptable (*α* = 0.81).

#### Communication

The Prerana Interview was adapted and utilized to assess patient/provider communication; it includes a 10-item measure asking about patient experience and feelings towards providers [28]. Attitudes towards treatment, patient-provider relationship, and psychosocial barriers to patient-provider communication are all measured by this assessment. Responses consist of a 4-point scale ranging from 0 (never) to 3 (every time). A sum score of this variable over time was used. Cronbach’s reliability coefficient for this scale was considered acceptable (*α* = 0.88).

#### Motivation for Adherence

The motivation subscale of the LifeWindows Information-Motivation-Behavioral Skills Adherence Assessment Questionnaire was used to measure motivation to be adherent to ART medication [29]. This tool is a 10-item assessment, utilizing a 5-point Likert scale ranging from 1 (strongly disagree) to 5 (strongly agree). The mean motivation score at each time point was used for analysis. Cronbach’s reliability coefficient for this scale was considered acceptable (*α* = 0.74).

#### Health Insurance Type

Having private health insurance was measured as a predictor with 0 indicating no health coverage and 1 indicating having private health coverage. As all people living in Argentina are eligible to receive universal healthcare, those who do not have private healthcare coverage receive coverage through the public healthcare system. For the purpose of this study, private healthcare insurance was considered as a predictor as previous literature has identified that the lack of private insurance, even when public insurance is available, is a barrier to healthcare [30].

### Statistical Analyses

Participants’ demographics and baseline characteristics were summarized using descriptive statistics. Mean and standard deviation (SD) were reported for continuous variables, whereas frequencies and percentages were reported for categorical variables.

A logistic regression model within the generalized linear mixed model (GLMM) framework was utilized to examine the predictors of self-reported adherence over time. The model accounted for fixed effects of time, self-efficacy, alcohol use, and other covariates, with random intercepts and slopes for subjects to capture individual variability in adherence trajectories. Interactions between time and predictors were included to assess how the associations changed over the study period. A Poisson regression model within the GLMM framework was applied to assess the predictors of the count of missed appointments with infectious disease doctors. This model also included time, average motivation, self-efficacy, alcohol use, and their interactions with time, along with random effects to account for individual differences. Similarly, a Poisson GLMM was employed to explore factors influencing missed clinic visits for lab work or medication pickup. The model incorporated time, private health insurance, alcohol use, communication, and their interactions with time as predictors, alongside random effects.

Analyses were conducted using R (version 2023.12.1 + 402 (2023.12.1 + 402)) [31, 32]. The lme4 package was used for fitting GLMMs [33]. The glmer function from lme4 was applied for logistic regression models, and glmer with a Poisson family was used for count data models.

## Results

### Sample Characteristics

Participants were an average of 40.07 years old (standard deviation (*SD*) = 10.96). Half (51%) of the participants were female, and 71% identified as heterosexual. Slightly more than half (56%) of participants were employed and 65% had completed high school or higher. Other demographic characteristics are described in Table 1. 17.8% of the participants did not complete an assessment from baseline to 6 months, of which 1.9% were deceased, 15% were missing or lost, and 0.8% withdrew. 25.6% of participants did not complete an assessment at 12 months (2.8% deceased, 20.8% missing or lost, and 1.9% withdrawn), 33.1% did not complete an assessment at 18 months (4.4% deceased, 26.7% missing or lost, and 1.9% withdrawn), and 26.7% did not complete an assessment at 24 months (4.7% deceased, 20% missing or lost, and 1.9% withdrawn).

**Table 1:** Sociodemographic characteristics of the sample (*N* = 360)

Variable	*N* (%) all
1. Age	
Range	19–79
Mean	40.07
2. Gender	
Men	174 (48.3%)
Women	183 (50.8%)
Other	1 (0.3%)
I do not want to answer	2 (0.6%)
3. Sexual orientation	
Heterosexual	257 (71.4%)
Homosexual	69 (19.2%)
Bisexual	16 (4.4%)
Other	1 (0.3%)
I do not know	6 (1.7%)
I do not want to answer	11 (3.1%)
4. Employment	201 (55.8%)
5. Education	
Completed less than high school	127 (35.3%)
Completed high school or more	233 (64.7%)
6. Nationality	
Argentinian National	333 (92.5%)
Migrant	27 (7.5%)

### Predictors of Self-reported Adherence Over Time

**Fig. 1:**
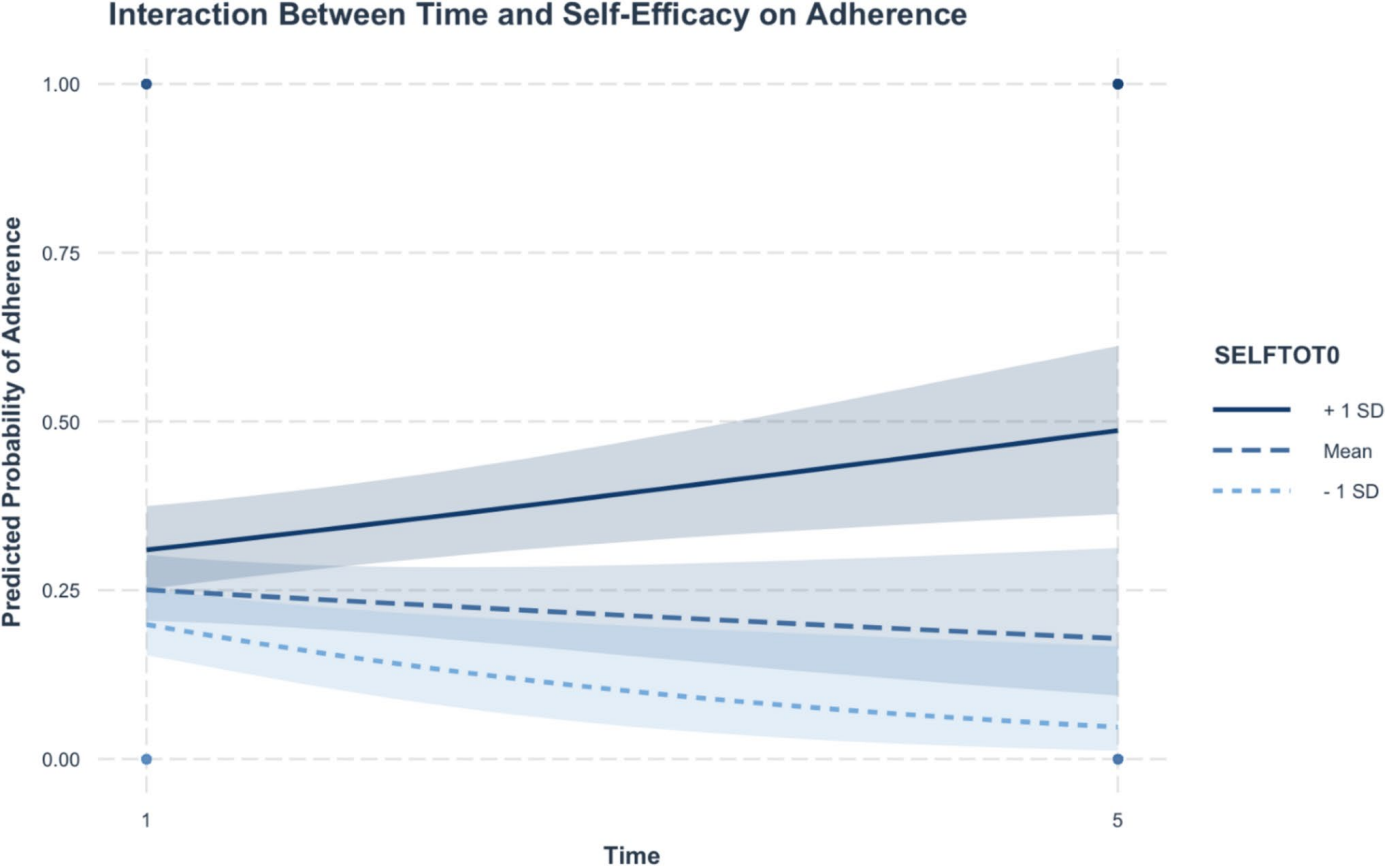
Probability of adherence over time by self-efficacy levels. *Note.* The solid, dashed, and dotted lines represent high (+ 1 SD), mean, and low (− 1 SD) levels of self-efficacy, respectively. The increasing trend for higher self-efficacy levels over time illustrates its positive impact on adherence. Shaded regions indicate confidence intervals

**Fig. 2:**
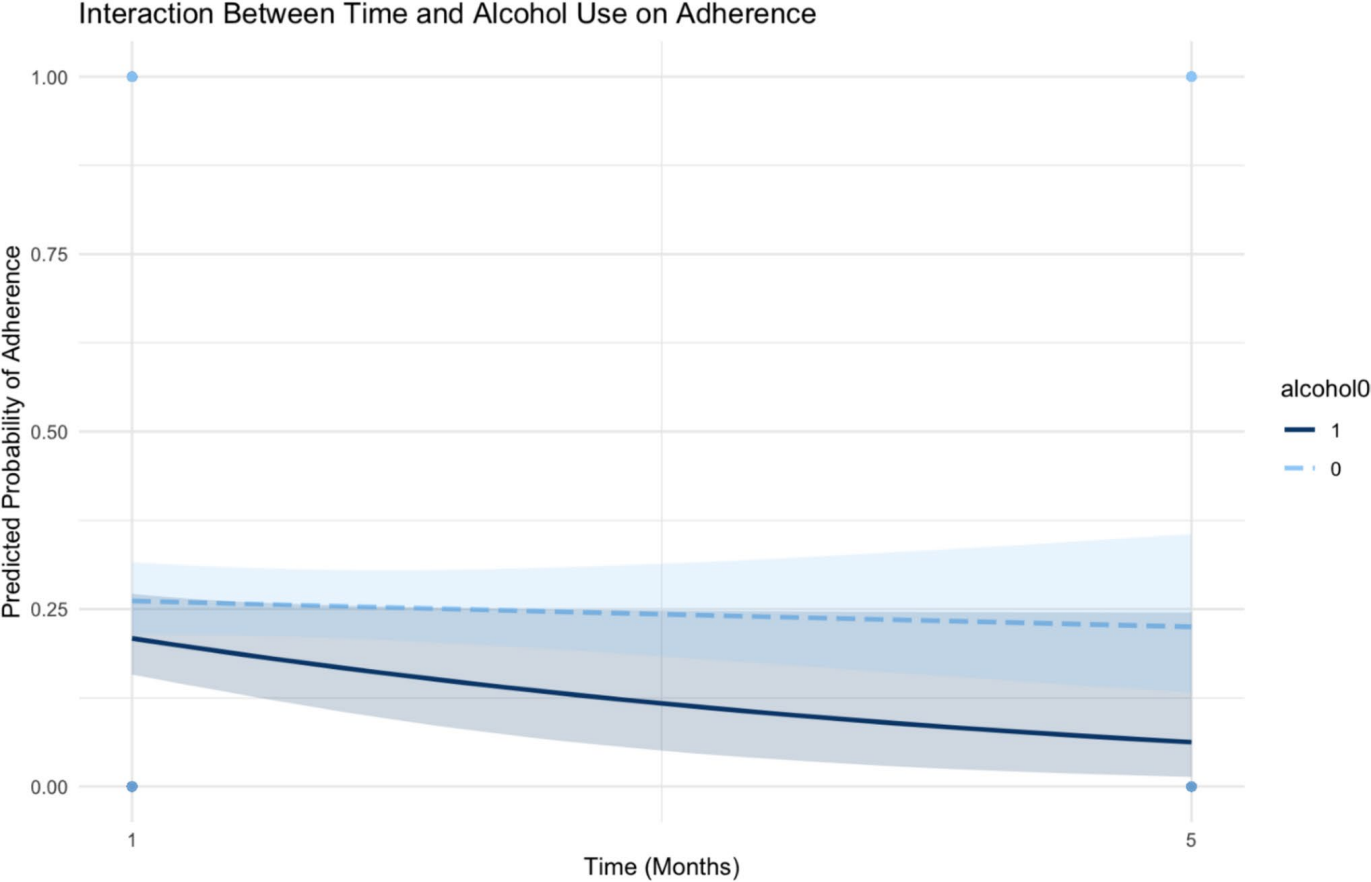
Interaction between time and alcohol use on adherence. *Note*. This plot illustrates the relationship between time, alcohol use, and predicted adherence to ART (antiretroviral therapy). Alcohol use (1 = Yes, 0 = No) is depicted as a moderating variable in the adherence trajectory over time. Participants who do not consume alcohol (dashed line) show relatively stable adherence rates, while those who consume alcohol (solid line) exhibit a declining adherence probability over time. The shaded regions represent 95% confidence intervals, highlighting the variability and uncertainty around the predictions. This emphasizes the negative impact of alcohol use on adherence behaviors over the study period

In examining factors influencing adherence, time significantly predicted the likelihood of decreased adherence, with each unit increase in time decreasing the odds of adherence outcomes by 77% (*B* = − 1.47, SE = 0.44, *z* = − 3.37, *p* = 0.001, OR = 0.23). Additionally, the interaction between self-efficacy and time was significant, indicating that higher self-efficacy scores were associated with slightly increased odds of adherence over time (*B* = 0.01, SE = 0.00, *z* = 3.88, *p* < 0.001, OR = 1.01), which is displayed in Fig. 1. Conversely, the interaction between alcohol use and time suggested that increased alcohol use was associated with reduced odds of adherence over time (*B* = − 0.29, SE = 0.14, *z* = − 2.16, *p* = 0.030, OR = 0.75). This interaction is depicted in Fig. 2. Other predictors, including demographic factors and healthcare needs, were not statistically significant. A summary of this model is provided in Table 2.

**Table 2:** Predictors of adherence based on a binomial generalized linear mixed-effects model

Predictor	*B* (SE)	*z*	*p*	Odds ratio (95% CI)
Intercept	0.47 (0.75)	0.63	0.526	1.60 (0.36, 7.08)
Time	− 1.47 (0.44)	− 3.37	***0.001	0.23 (0.10, 0.52)
Self-perceived gender	0.02 (0.05)	0.35	0.724	1.02 (0.92, 1.13)
Age	− 0.45 (0.25)	− 1.83	0.068	0.64 (0.41, 1.00)
National-immigrant	− 0.43 (0.39)	− 1.11	0.265	0.65 (0.30, 1.40)
Education level	− 0.30 (0.23)	− 1.31	0.191	0.74 (0.46, 1.19)
Financial support state	− 0.30 (0.33)	− 0.92	0.360	0.74 (0.39, 1.40)
Private health insurance	0.17 (0.21)	0.80	0.422	1.19 (0.76, 1.85)
Complex healthcare needs	− 0.06 (0.19)	− 0.30	0.762	0.94 (0.65, 1.36)
Average motivation × time	0.00 (0.04)	0.07	0.941	1.00 (0.96, 1.05)
Self-efficacy × time	0.01 (0.00)	3.88	*** < 0.001	1.01 (1.01, 1.02)
Alcohol × time	− 0.29 (0.14)	− 2.16	*0.030	0.75 (0.59, 0.95)
Communication × time	0.01 (0.01)	1.41	0.159	1.01 (0.99, 1.04)

*B*, log-odds estimate; *SE*, standard error; odds ratios are calculated as exp(*B*). 95% confidence intervals for odds ratios are not provided due to space constraints but can be calculated as exp(*B* ± 1.96 × SE). *p < 0.05. **p < 0.01. ***p < 0.001

### Predictors of Missed Infectious Disease Appointments Over Time

Analysis of factors influencing missed appointments with infectious disease doctors revealed significant predictors. Time emerged as a significant predictor, indicating that for each additional time unit, the rate of missed appointments increased by 57% (*B* = 0.45, SE = 0.12, *z* = 3.70, *p* < 0.001, IRR = 1.57) (Table 3). Additionally, the interaction effects of time with motivation, self-efficacy, alcohol use, and communication were significant. Specifically, an interaction between time and average motivation was associated with a 6% decrease in the rate of missed appointments per time unit (*B* = − 0.07, SE = 0.03, *z* = − 2.30, *p* = 0.021, IRR = 0.94). Similarly, time interacting with self-efficacy (*B* = − 0.002, SE = 0.001, *z* = − 2.20, *p* = 0.028, IRR = 0.998) and alcohol use (*B* = 0.11, SE = 0.05, *z* = 2.19, *p* = 0.029, IRR = 1.12) demonstrated significant effects on missed appointments.

**Table 3:** Predictors of missed appointments with an infectious disease doctor based on a generalized linear mixed-effects model (Poisson family)

Predictor	*B* (SE)	*z*	*p*	IRR (95% CI)
Intercept	0.50 (0.52)	0.97	0.334	1.65 (0.63, 4.32)
Time	0.45 (0.12)	3.70	***0.0002	1.57 (1.23, 2.00)
Self-perceived gender	− 0.02 (0.04)	− 0.46	0.649	0.98 (0.90, 1.07)
Age	0.32 (0.17)	1.89	0.059	1.38 (0.99, 1.92)
National-immigrant	− 0.23 (0.29)	− 0.78	0.435	0.80 (0.48, 1.33)
Education level	− 0.08 (0.17)	− 0.51	0.612	0.92 (0.65, 1.30)
Financial support state	0.22 (0.20)	1.07	0.284	1.25 (0.84, 1.85)
Private health insurance	− 0.20 (0.16)	− 1.24	0.216	0.82 (0.61, 1.10)
Complex healthcare needs	− 0.02 (0.14)	− 0.14	0.887	0.98 (0.74, 1.30)
Average motivation × time	− 0.07 (0.03)	− 2.30	*0.021	0.94 (0.89, 0.98)
Self-efficacy × time	− 0.002 (0.001)	− 2.20	*0.028	0.998 (0.997, 0.999)
Alcohol × time	0.11 (0.05)	2.19	*0.029	1.12 (1.01, 1.25)
Communication × time	− 0.01 (0.004)	− 2.65	**0.008	0.99 (0.98, 1.00)

*B*, log-odds estimate; *SE*, standard error; *IRR*, incidence rate ratio, calculated as exp(*B*). 95% confidence intervals for IRRs are calculated as exp(*B* ± 1.96 × SE). *p < 0.05. **p < 0.01. ***p < 0.001

### Predictors of Missed Clinic Appointments Over Time

Investigations into the predictors of missed clinic visits for lab work or medication pickup using a Poisson generalized linear mixed-effects model revealed several key factors (Table 4). The model showed a significant increase in missed visits over time (*B* = 0.28, SE = 0.13, *z* = 2.11, *p* = 0.035, IRR = 1.32). Notably, having private health insurance significantly reduced the rate of missed visits, with an IRR of 0.494, indicating that individuals with private health insurance were about half as likely to miss clinic visits compared to those without (*B* = − 0.705, SE = 0.149, *z* = − 4.75, *p* < 0.001). Additionally, interactions between time and alcohol use (*B* = 0.113, SE = 0.056, *z* = 2.02, *p* = 0.044, IRR = 1.12) and time and communication (*B* = − 0.013, SE = 0.004, *z* = − 3.28, *p* = 0.001, IRR = 0.987) were significant, indicating that these factors influence on clinic visit adherence changed over time.

**Table 4:** Predictors of missed clinic visits based on a generalized linear mixed-effects model (Poisson family)

Predictor	*B* (SE)	*z*	*p*	IRR (95% CI)
Intercept	1.34 (0.47)	2.82	**0.005	3.81 (1.47, 9.89)
Time	0.28 (0.13)	2.11	*0.035	1.32 (1.02, 1.71)
Self-perceived gender	− 0.003 (0.037)	− 0.08	0.934	0.997 (0.926, 1.073)
Age	− 0.235 (0.160)	− 1.47	0.143	0.790 (0.634, 0.986)
National-immigrant	0.155 (0.270)	0.57	0.566	1.168 (0.690, 1.975)
Education level	− 0.145 (0.149)	− 0.97	0.331	0.865 (0.633, 1.183)
Financial support state	− 0.036 (0.192)	− 0.19	0.852	0.964 (0.658, 1.412)
Private health insurance	− 0.705 (0.149)	− 4.75	*** < 0.001	0.494 (0.350, 0.697)
Complex healthcare needs	0.188 (0.122)	1.54	0.124	1.207 (0.955, 1.524)
Average motivation × time	− 0.048 (0.030)	− 1.58	0.114	0.953 (0.898, 1.011)
Self-efficacy × time	− 0.001 (0.001)	− 1.52	0.129	0.999 (0.997, 1.000)
Alcohol × time	0.113 (0.056)	2.02	*0.044	1.120 (1.005, 1.248)
Communication × time	− 0.013 (0.004)	− 3.28	**0.001	0.987 (0.979, 0.995)

*B*, log-odds estimate; *SE*, standard error; *IRR*, incidence rate ratio, calculated as exp(B). 95% confidence intervals for IRRs are calculated as exp(*B* ± 1.96 × SE). *p < 0.05. **p < 0.01. ***p < 0.001

## Discussion

This study examined the impact of time and self-efficacy, communication, motivation, alcohol, and health insurance type on treatment adherence and attendance among individuals lost to care being re-engaged in HIV care in Argentina. As in previous studies of chronic illness, time was associated with lower self-reported adherence and more missed infectious disease visits and missed lab appointments in this study sample. The interaction of self-efficacy and time was associated with increased likelihood of adherence over time while, in contrast, the interaction of time and alcohol use was associated with a decreased likelihood of adherence and increased missed ID and general lab/clinic appointments over time. The interaction of time and motivation and self-efficacy was associated with fewer missed ID specialist appointments over time, and the interaction of communication and time was associated with decreased rate of missed lab/clinic visits. Having private health insurance was associated with better adherence and attendance.

The results of the current study support existing literature on the association between alcohol use and adherence. Those with a history of alcohol use were more likely to miss clinic appointments and not adhere to medication [34–36]. Not only is alcohol use associated with non-adherence, but also the incidence and course of HIV [37] and is considered a major risk factor for morbidity and mortality in Latin America [38]. As problematic drinking is underrepresented in HIV research on adherence in Latin American countries and may be culturally acceptable in Latin American societies, its impact on adherence should be addressed in future studies [39].

Additionally, the association between increased self-efficacy and an increased likelihood of adherence and visit attendance has been investigated and is supported by the current study findings [15, 40]. Prior research has shown that patient-provider communication can increase self-efficacy and motivation, and in turn, promote adherence [40]. Self-efficacy and motivation can also mediate the effect between mental health issues and appointment attendance and adherence, making them important components of future interventions [41]. Self-efficacy may also play an important role in mediating the effect of stigma on ART adherence, which is especially important to consider in interventions addressing minority populations who may experience increased stigma [42].

The results of the current study further highlight the importance of positive communication with healthcare providers and should be addressed in future interventions targeting adherence and retention. The association between positive communication and treatment adherence and fewer missed lab appointments is also well documented in the literature [43]. Some previous research suggests that communication training should be provided to not only physicians, but also nurses and other healthcare workers [44]. Additionally, as some patients may prefer certain types of patient-provider communication, researchers should focus on developing and tailoring specific communication styles to best serve the patient and ensure adherence and retention in care [45]. Other clinical interventions that may address psychosocial aspects of the lack of treatment adherence, such as cognitive behavioral therapy and motivational interviewing [46], have been beneficial in increasing adherence and reducing viral load, and motivational interviewing has been successfully implemented by HIV providers for this population in Argentina [47]. In fact, other research has illustrated that patients who completed visits with HIV clinicians using motivational interviewing rated their experiences as more positive and patient centered [48]. Moreover, providing MI training to HIV providers may enhance patient-provider communication and self-efficacy, therefore increasing treatment adherence. Finally, the impact of the current factors discussed in this study on not only ART adherence, but also on HIV risk among sexual minority men, is important to address in the context of investigating the effects of minority stress in these populations [49].

Previous research has shown an association between private clinic attendance and adherence and has emphasized the positive effects of certain measures employed in private healthcare clinics [16]. For instance, private clinic treatments consist of at least 2–3 visits per year usually with the same ID specialist and have a computerized follow-up system for missed appointments and prescription refills, while in public clinics, patients may not see the same specialist at each appointment and patients are expected to initiate follow-up [50]. Overall, patients take less of an active role in private clinics as many services are initiated and offered to the patients in one location [16]. Furthermore, interventions should consider emulating private clinic practices in public clinics to improve adherence and clinic attendance.

### Limitations and Future Research

While this study offers valuable insights, it is not without its limitations. The reliance on self-reported adherence measures may introduce bias. Future studies could benefit from the inclusion of objective adherence measures, such as pharmacy refill data or electronic adherence monitoring. Implementing retention strategies in study activities should also be considered to limit the number of participants lost to follow-up that may affect future studies of adherence among PLWH. Additionally, the study’s focus on urban centers in Argentina may limit the generalizability of the findings to rural populations, highlighting the need for research in these areas. For instance, healthcare access, whether private or public, is more accessible in urban areas and results from this study do not account for disparities in access to HIV care in rural communities [51]. Despite these limitations, the dynamic nature of the associations between communication, self-efficacy, motivation, alcohol use, insurance, and treatment adherence over time points is a rich area for future research. Longitudinal studies that further dissect these associations can offer deeper insights into how interventions can be tailored over the course of treatment to maximize adherence. Moreover, exploring the role of digital health interventions, such as mobile health apps designed to enhance self-efficacy and motivation, could offer innovative solutions to the adherence challenges highlighted in this study, particularly attrition.

## Conclusion

Findings from this study provide insight into the impact of patient-provider communication, patient self-efficacy, motivation, and alcohol use and insurance, among Argentine individuals living with HIV who are lost to care. ART adherence and patient engagement are essential components of optimizing patient outcomes and reducing the risk of HIV transmission [52]. The association between alcohol use and treatment adherence highlights the need for interventions targeting alcohol use and mental health. Moreover, the role of self-efficacy, communication, and motivation may play a crucial and interconnected role in adherence and should be addressed in future public health interventions to cultivate positive patient/provider relationships.

## Data Availability

Data is available upon request from the corresponding author.

## Declarations

### Welfare of Animals

This article does not contain any studies with animals performed by any of the authors.
